# From a Chemotherapeutic Drug to a High-Performance Nanocatalyst: A Fast Colorimetric Test for Cisplatin Detection at ppb Level

**DOI:** 10.3390/bios12060375

**Published:** 2022-05-30

**Authors:** Valentina Mastronardi, Mauro Moglianetti, Edoardo Ragusa, Rodolfo Zunino, Pier Paolo Pompa

**Affiliations:** 1 Nanobiointeractions & Nanodiagnostics, Istituto Italiano di Tecnologia (IIT), Via Morego, 16163 Genova, Italy; valentina.mastronardi@iit.it (V.M.); mauro.moglianetti@ismn.cnr.it (M.M.); 2 Department of Electrical, Electronic, Telecommunications Engineering and Naval Architecture, University of Genova, Via Opera Pia, 11a, 16145 Genova, Italy; edoardo.ragusa@edu.unige.it (E.R.); rodolfo.zunino@unige.it (R.Z.)

**Keywords:** point-of-care, cisplatin, nanocatalyst, colorimetric test, machine learning

## Abstract

A rapid point-of-care method for the colorimetric detection of cisplatin was developed, exploiting the efficient conversion of the chemotherapeutic drug into a high-performance nanocatalyst with peroxidase enzyme mimics. This assay provides high specificity and ppb-detection sensitivity with the naked eye or a smartphone-based readout, outperforming many standard laboratory-based techniques. The nanocatalyst-enabled colorimetric assay can be integrated with machine-learning methods, providing accurate quantitative measurements. Such a combined approach opens interesting perspectives for the on-site monitoring of both chemotherapeutic patients to achieve optimal treatments and healthcare workers to prevent their unsafe exposure.

## 1. Introduction

Platinum-based chemotherapy drugs, such as cisplatin, are extensively used for the treatment of several types of cancer [1,2,3,4], either as single agents or in combination with other antineoplastic drugs [5,6,7,8]. Cisplatin is, however, highly toxic and can induce severe side effects in treated patients, including nausea, ototoxicity, neurotoxicity, and serious kidney damage, which partly limits its clinical efficacy [9,10,11]. Therefore, the monitoring of cisplatin in patients’ body fluids, such as in blood or urine, would be fundamental to study dose-related pharmacokinetics and toxicodynamics, achieving optimal treatment and efficacy. Moreover, emerging evidence reveals cisplatin can be highly detrimental to healthcare workers, who are daily exposed during the preparation and/or administration of chemotherapy [12,13,14]. The continuous work-related exposure to cisplatin might cause various organ damage and severe renal toxicity [13,15,16,17,18]. Other chronic effects linked with exposure to antineoplastic drugs, including spontaneous abortion and congenital anomalies [18,19,20,21], have also been reported. Considering such toxicological profile and the possible long-term health effects, it would be crucial to frequently monitor environmental contaminations, to prevent unsafe handling of chemotherapeutics and their consequent dispersion, with the aim of safeguarding healthcare workers. However, for both cancer patients and healthcare workers, the continuous monitoring of cisplatin levels in biological fluids or as surface contaminations is hampered by the time-consuming and lengthy procedures required by state-of-the-art technology, relying on complex instrumentation and centralized laboratories. To date, the reference analytical techniques are atomic absorption spectrometry [22,23], inductively coupled plasma mass spectrometry [22,24,25], stripping voltammetry [24,25,26], HPLC coupled to different kinds of detectors [27,28,29,30], and/or electrochemical methods [31,32,33], whereas fast and on-site sensing methods would represent a key technological advancement. To address such issues, some alternative approaches based on aptamers and biosensors have been recently explored, although they do not completely overcome the use of complex procedures and instrumentations, such as electrochemical stations, spectrophotometers, quartz crystal microbalances, or sequencing machines [32,34,35,36,37,38,39]. In this work, we developed a point-of-care (POC) strategy for frequent on-site analyses of cisplatin with high sensitivity. We introduced a method harnessing the quick reduction of the chemotherapeutic drug to form small platinum nanoparticles, which are then exploited to efficiently catalyze a chromogenic reaction with consequent color generation, detectable by the naked eye. This strategy allows a fast, specific, and instrument-free detection of cisplatin with ppb sensitivity, outperforming many laboratory-based techniques.

## 2. Materials and Methods

### 2.1. Reagents and Equipment

Cis-diammine platinum dichloride, hydrochloric acid puriss. p.a., ACS reagent, reag. Ph. Eur., ≥37%, sodium citrate tribasic dehydrate BioUltra, sodium borohydride, and citric acid anhydrous were purchased from Merck/Sigma-Aldrich (Darmstadt, Germany) and used as received. Hydrogen peroxide (H_2_O_2_) solution was obtained from Sigma-Aldrich (Darmstadt, Germany). 3,3′,5,5′-tetramethylbenzidine (TMB) solution was purchased from Kementec (Taastrup, Denmark). All chemicals were used as received. All solutions were prepared with distilled, deionized water (Millipore, Milli-Q system, Darmstadt, Germany) with a resistivity of 18.2 MΩ·cm. UV-vis measurements were performed by using NanoDrop OneC spectrophotometer (ThermoFisher Scientific, Waltham, MA, USA) equipped with a 1 cm path length cell.

### 2.2. Preparation of Cisplatin

The stock solution of cisplatin was obtained by dissolving 1 mg of the reagent in 1 mL of HCl at 3.3 mM. To obtain a good dispersion, the solution was kept 15 min at 80 °C. Immediately after this step, the stock solution was diluted at different concentrations ranging from 0 to 1600 ppb.

### 2.3. Synthesis of PtNPs from Cisplatin Precursor

PtNPs were synthesized by adding 954 µL of diluted solution of cisplatin in a 2-mL glass vial immediately followed by a quick addition of 24 µL solution containing sodium citrate and citric acid (at 0.03 M and 2 mM concentration, respectively) and 22 µL of freshly prepared NaBH_4_ (0.02 M). The vial was moved into a thermoblock at 100 °C to obtain a quick reduction of the Pt ions. The reaction time was 10 min.

### 2.4. Synthesis of PtNPs from Cisplatin Precursor with Pt Seed Baseline

PtNPs were synthesized by adding in a 2-mL glass vial 100 µL of Pt seeds (3 nm size, 20 ppb concentration), 854 µL of diluted solution of cisplatin followed by the addition of the solution containing sodium citrate and citric acid and freshly prepared NaBH_4_, as described above. The vial was moved into a thermoblock for 10 min at 100 °C to obtain seed-mediated growth.

### 2.5. Synthesis of PtNPs from Cisplatin Precursor with Chloroplatinic Acid Hexahydrate Baseline

PtNPs were synthesized by adding in a 2-mL glass vial 100 µL of aqueous solution of chloroplatinic acid hexahydrate (at 2.3 µM), 854 µL of diluted solution of cisplatin, and previously described solutions containing sodium citrate and citric acid and freshly prepared NaBH_4_. The vial was placed into a thermoblock at 100 °C for 10 min to obtain a quick reduction of the Pt ions. The characterization of the PtNPs obtained is the same mentioned above.

### 2.6. Synthesis of PtNPs in Synthetic Urine

A stock solution of synthetic urine was prepared as reported by Sarigul et al. [40]. Then, 900 µL of this solution was mixed with 100 µL of aqueous solution containing chloroplatinic acid hexahydrate (at 2.3 µM) and cisplatin (at 4.4 or 8.5 µM for 850 and 1650 ppm concentration, respectively). The solution of the synthetic urine and precursors was diluted 50 times before use. PtNPs were synthesized by adding 954 µL of the diluted solution in a 2-mL glass vial followed by the addition of the solution containing sodium citrate and citric acid and freshly prepared NaBH_4_. The reduction of Pt ions was obtained with the same method described for PtNPs from cisplatin precursor.

### 2.7. PtNP Characterization

The characterization of the PtNPs was performed by transmission electron microscopy (TEM) by using a JEOL JEM 1011 microscope (Genoa, Italy), after deposition on carbon-coated grids. The diameter of PtNPs was obtained by applying a threshold on the BF-TEM images, followed by automatic measurement by using ImageJ.

### 2.8. Colorimetric Method

A total of 167 µL of TMB solution (used as received from Kementec) and 267 µL of 2 M H_2_O_2_ were quickly added to the previously obtained PtNP suspension at pH 6 in water and pH 6.5 in synthetic urine. After 5 min at room temperature, the color change was quantified by measuring the absorbance at 652 nm by NanodropC spectrophotometer. Water was used as a negative control.

### 2.9. Machine Learning

A dataset with 62 test tubes has been prepared with fixed lighting and framing. The dataset contains 22 test tubes with concentration lower or equal to 65 ppm, covering values 1.5, 3, 6.5, 17, 33, 45, 65. The remaining 40 test tubes were equally divided between the following concentrations: 100, 200, 350, 500, 600, 700, 900, 1000, 1100, and 1200. The recording lasts for 150 sec, 1 frame per second. The Knn K parameter was set to 1. The time series are preprocessed by using a moving average with a five-second window and 5 s stride leading to time series with 30 samples. The distances were measured as the average distance computed over the channels. A holdout procedure assessed the performance of the algorithm. In particular, a leave-one-out procedure was employed.

## 3. Results

### 3.1. Nanozyme-Based Method for the Detection of Cisplatin

**Figure 1:**
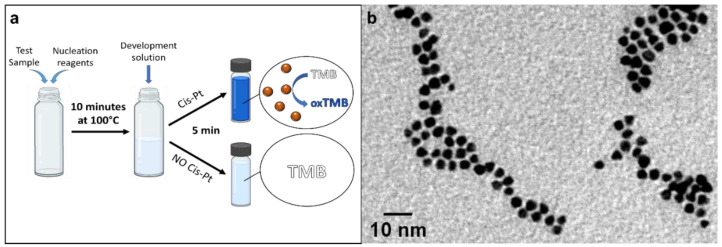
(**a**) Scheme of the two-step colorimetric method for the detection of cisplatin. In the presence of the chemotherapeutic drug, the reducing agent quickly promotes the formation of small (ca. 4 nm diameter) monodisperse PtNPs, which in turn catalyze the oxidation of the TMB chromogen, with consequent formation of a visible blue color. In the absence of cisplatin contaminations, the solution remains transparent. (**b**) Representative TEM image of the PtNPs formed from cisplatin.

The POC method here proposed for the rapid and visual evaluation of cisplatin contaminations is described in Figure 1a. The underlying principle of cisplatin sensing relies on the use of the chemotherapeutic drug as a precursor for the fast growth of Pt nanoparticles, a highly catalytic nanomaterial [41,42]. In particular, PtNPs are very efficient peroxidase enzyme mimics, able to catalyze the oxidation of a chromogenic probe (e.g., TMB) into an intense blue-colored compound [43,44,45,46,47]. Thanks to a careful design of the reaction conditions and stabilizing agents, leading to the formation of small nanoparticles with high surface-to-volume ratio, such a process enables the visual detection of an extremely low concentration of cisplatin in only 15 min, without the use of any instrument, apart from a small and portable heating block. Moreover, the method provides high specificity toward platinum species, offering a clear optical readout, detectable either by the naked eye or by a standard smartphone camera for quantitative measurements. Our strategy is based on two main steps (Figure 1a). In the first phase of the assay, the use of a reducing agent (NaBH_4_) rapidly elicits the formation of Pt(0) species from cisplatin, followed by their nucleation and final growth of Pt nanoparticles (PtNPs) stabilized by citrate capping (see Methods for details). Optimizing the synthesis conditions, we managed to produce very small PtNPs of ca. 4.5 ± 0.7 nm, in the whole concentration range tested (Figure 1b and Figure S1, see the Supplementary Materials), boosting their surface-to-volume ratio (namely the catalytic activity per Pt mass unit) and thus the overall sensitivity of the POC method. In addition, the reaction is completed in only 5–10 min. The accurate control of the particle size along with the low size dispersion were achieved through the interplay of temperature and sodium borohydride. In particular, the concentration of the reducing agent plays a crucial role in determining the final size of the nanomaterial. Low concentrations of NaBH_4_ were not sufficient to promote the growth of the NPs (losing cisplatin detection sensitivity), whereas high concentrations favored particle aggregation and polydispersity (decreasing the available catalytic surface) (see Figure S2). Indeed, although NaBH_4_ is necessary for the formation of PtNPs, an excess of its concentration is deleterious for the reaction, promoting uncontrolled particle growth and resulting in the formation of large polydisperse particles. Therefore, NaBH_4_ was used at a high temperature (100 °C) and at a concentration guaranteeing a quick controlled reaction and resulting in its complete consumption (thus preventing possible interference during the second step of the sensing scheme). On the other side, the citrate molecules in solution act as the particle capping agents to stabilize the NPs. Unlike other surface shielding ligands (i.e., polymers or other sticky surface coatings, such as PVP, which limit the particle surface accessibility to catalytic reactions), citrate also maximizes the catalytic performance of the formed particles [48,49,50,51,52] and, in turn, the detection sensitivity. In the second step, the formed PtNPs, specifically derived from the presence of cisplatin, are detected by adding a color-development solution, containing a chromogenic substrate (TMB) and hydrogen peroxide. The color change is based on the reaction of H_2_O_2_ and TMB catalyzed by the Pt nanozymes acting as peroxidase mimics. In particular, H_2_O_2_ is first activated on the particle surface, leading to the formation of the OH• radical [53,54,55,56], eliciting the oxidation of the TMB substrate [54,55] with the release of water and formation of a colored TMB compound [44,57,58]. The fast generation of a blue color (<5 min), detectable by the naked eye, is then indicative of the presence of cisplatin, whereas a transparent solution indicates a cisplatin-free sample.

### 3.2. Selectivity and Sensitivity of the Detection Scheme

**Figure 2:**
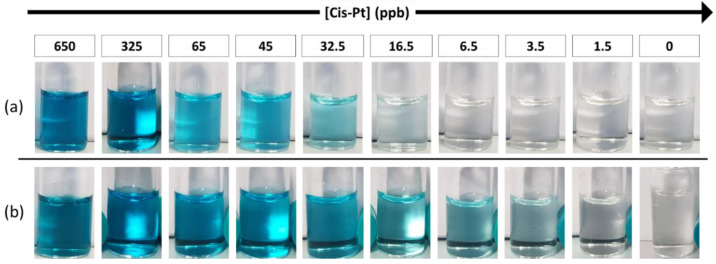
Representative colorimetric results of the assay, after 5 min color development. (**a**) Color change obtained at different concentrations of cisplatin (LOD ~30 ppb) in the standard configuration. (**b**) Colorimetric results in the enhanced configuration of the test (LOD ~3 ppb).

This detection scheme has been designed to be highly sensitive and selective, because PtNPs are formed only in the presence of cisplatin in solution, eliciting the oxidation of the TMB and the typical color change from transparent to blue. A representative photograph of the colorimetric assay is reported in Figure 2a. Upon careful design of the various reaction parameters and sizing the PtNPs around 4 nm, we achieved an excellent detection sensitivity for cisplatin, with a visual limit of detection (LOD) around 30 ppb. This is also due to the very efficient reduction of cisplatin to Pt(0) during the initial phase of the reaction with subsequent formation of PtNPs. Interestingly, under identical conditions, cisplatin was found to be a significantly more efficient precursor for the formation of PtNPs than the reference hexachloroplatinic acid, which has a higher oxidation state (see Figure S3). However, despite such very good detection limit by an instrument-free readout, we were able to further improve it, by introducing a sensitivity enhancement strategy. Specifically, we relied on the addition of a very low Pt concentration in the first step of the reaction, with the aim of boosting the assay sensitivity without causing any interference in absence of cisplatin (no false positives). To this purpose, we designed two experiments, probing either a Pt salt or Pt seeds. In the latter setup, we tried to take advantage of a seed-mediated growth method to decrease the LOD. Cisplatin was reduced directly on the surface of the Pt seeds previously inserted in the solution, allowing their growth to form larger nanoparticles. With this method, a LOD of 7 ppb of cisplatin was reached (Figure S4). In this configuration, to avoid false positive results, a very low concentration of Pt seeds in solution must be used, because of the catalytic properties of the starting material itself, which limits the overall sensitivity gain. On the other hand, we probed the performance of Pt ions (hexachloroplatinic ions, PtCl_6_^2−^) as the Pt species baseline. Interestingly, the combination between cisplatin and hexachloroplatinic ions (which have no catalytic activity alone) could overcome the sensitivity limit, achieving an outstanding naked-eye LOD of ca. 3 ppb (Figure 2b), which is comparable to or even better than many instrumental techniques, such as UV-vis spectrophotometers and ICP-OES (see, for instance, Figure S5). Hexachloroplatinic ions are stable in water and are an ideal reagent to promote controlled NP growth in presence of cisplatin traces, leading to a significant sensitivity gain (ca. 1-order of magnitude). Under the optimized conditions, in absence of cisplatin, the selected concentration of hexachloroplatinic ions did not cause the formation of PtNPs, so no colorimetric signals were detected in the negative controls (Figure 2b and Figure S4).

**Figure 3:**
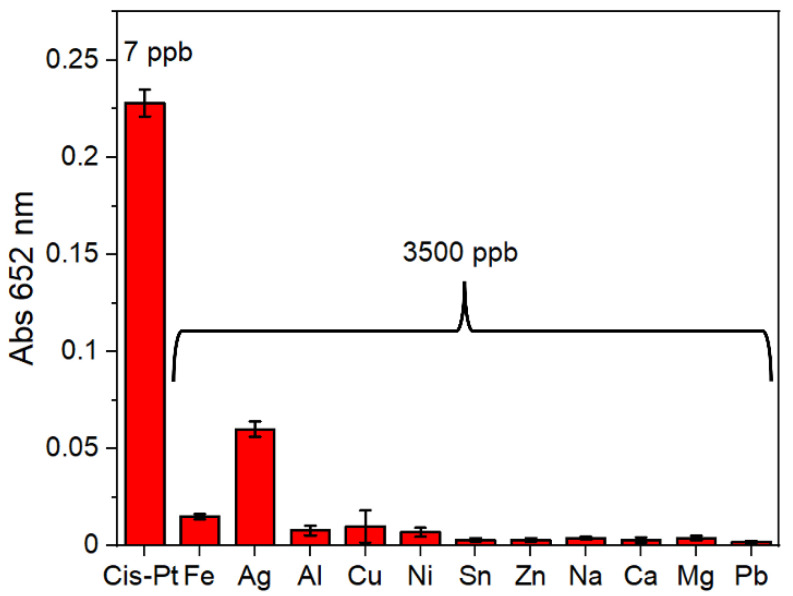
Selectivity of the colorimetric assay with respect to other toxic metal cations, in terms of absorbance signal measured after 5 min of reaction. Results are expressed as mean ± SD of tripled experiments.

After we maximized the test sensitivity, we proved its specificity, because real samples could contain various metal cations that need to be discriminated by the assay to avoid false positive results. We thus analyzed the selectivity of the proposed method, by evaluating the effect of different ions, at a concentration 1000 times higher than the test LOD. As shown in Figure 3, only cisplatin could induce a clear colorimetric response, with a significant increase in absorption at 652 nm (corresponding to the generation of an intense blue color), unlike the other cations analyzed, which did not produce any significant spectral changes, with the relative solutions remaining transparent. Only in the case of silver ions, a slight increase in the absorbance value occurred, but only for very high concentrations (3500 ppb), extremely unlikely in biological fluids or in hospital environment. The high selectivity of the test is due to the unique capacity of cisplatin, with respect to other metals tested herein, to combine the efficient formation of nanoparticles with their very high peroxidase activity generating the colorimetric readout.

### 3.3. Cisplatin Detection in Urine Matrix

**Figure 4:**
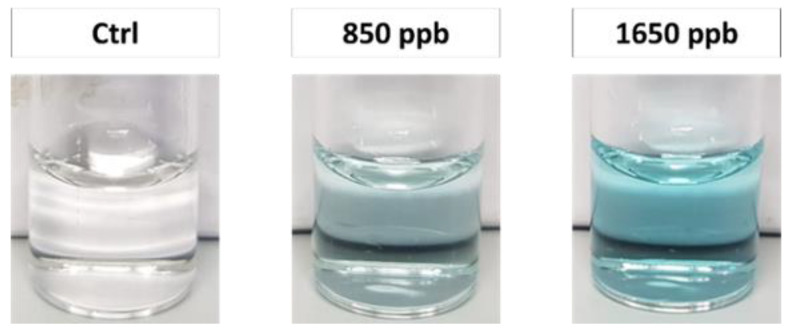
Color change obtained after 5 min at two different concentrations of cisplatin vs. negative control, in diluted synthetic urine (1:50).

As mentioned above, this POC method would be very advantageous to rapidly monitor the pharmaco-dynamics of cisplatin through the continuous analysis of the drug concentration in the biofluids of patients. During chemotherapeutic treatment, the concentration of cisplatin in the urine is known to range from an initial peak value (~1.2 ppm) down to below 0.4 ppm [53]. Here, as a preliminary proof-of-principle test, we evaluated the suitability of the nanocatalyst-mediated colorimetric assay to work in spiked samples of synthetic urine [54], without complex pre-analytical steps, such as purification. Urine, like other biological fluids, has a complex matrix that may cause interferences and/or signal attenuation. Interestingly, our method was proven to also be specific in urine samples, with no false positive results. Taking advantage of the high sensitivity of the assay, a simple pre-dilution of urine was sufficient to guarantee colorimetric detection of cisplatin around the 0.8 ppm level, namely in the clinically relevant range during treatments (Figure 4). Moreover, for urine samples, the blue color generation was clearly detectable by the naked eye after only 5 min. This POC approach is thus promising as a novel clinical tool for patient monitoring, although a systematic clinical validation and further improvements/optimizations are still necessary before its possible use in diagnostic applications.

### 3.4. Machine-Learning Analysis

The proposed method allows very sensitive cisplatin detection by simple visual inspection, yet the optical readout can only provide a qualitative ON/OFF response. However, several applications, especially in patient treatment monitoring, would strongly benefit from quantitative cisplatin measurements in biological fluids. Hence, with the aim of combining accurate quantitative analyses with POC characteristics for fast on-site screenings, we designed and developed a quantitative approach relying on machine-learning (ML) methods, working with very basic video cameras (thus being also suitable for application with standard smartphones). In particular, our ML approach exploited the kinetic information of the colorimetric reaction and a combination of dynamic time warping (DTW) [59,60,61,62] with the K-nearest neighbors (KNN) algorithm [63,64,65], allowing the use of a very limited experimental dataset. The details of the ML procedure are reported in Supporting Information and Materials and Methods.

**Figure 5:**
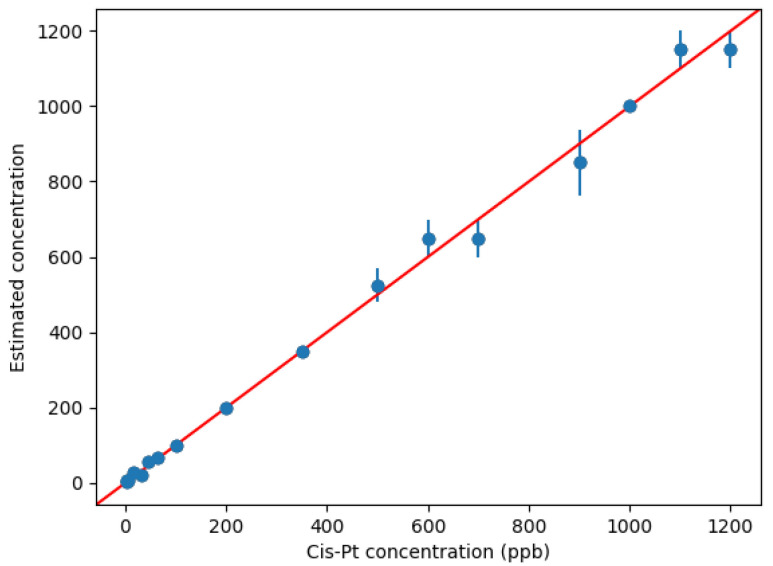
Quantitative measurements by ML: on the *y*-axis, the values predicted by the ML method are shown against the experimental concentrations of cisplatin (on the *x*-axis). For each concentration, the average value of the prediction is displayed by using a dot (the bars indicate the corresponding standard deviations). The red line sets the reference for the correct prediction.

The results obtained by the KNN/DTW method showed that such approach can provide accurate quantitative measurements of cisplatin concentrations, based on our colorimetric technique (Figure 5). In particular, Figure 5 reveals that the average predictions were always close to the ideal reference (red line) in the whole concentration range analyzed. Notably, the quantitative predictions in the low concentration range (<400 ppb) were extremely accurate with practically negligible errors. At a higher concentration, relatively larger errors were found, but always lower than 10%, which is an excellent result for a POC system. Indeed, the algorithm never assigned a concentration level that was distant more than one of the two neighbor concentrations inside the dataset. The experiments confirmed that DTW is an excellent distance measure for the kinematic time series [59]. In combination with KNN, the results highlighted a very good estimation of the concentration level even in presence of a dataset with a limited number of samples. Accordingly, the proposed solution can support quantitative measurements of the cisplatin by using the novel POC method proposed in this paper.

## 4. Conclusions

In conclusion, in this work we have reported a very sensitive (3 ppb LOD) colorimetric method for fast on-site analyses of cisplatin levels, exploiting the efficient and rapid synthesis of PtNPs from a cisplatin precursor and their superior catalytic properties for the fast oxidation of a chromogenic substrate. The whole assay can be performed in only 15 min, without the use of any complex instrument. The development of a reliable machine-learning method allows accurate quantitative measurements, opening interesting perspectives for the monitoring of both chemotherapeutic patients and healthcare workers.

## Data Availability

Not applicable.

## Supplementary Materials

The following supporting information can be downloaded at: https://www.mdpi.com/article/10.3390/bios12060375/s1, Figure S1: Size distributions of PtNPs obtained with different concentrations of cisplatin; Figure S2: BF-TEM images of aggregated PtNPs obtained with a high concentration NaBH_4_; Figure S3: Comparison of 230 nM of cisplatin and chloroplatinic acid; Figure S4: Comparison of sensitivity using chloroplatinic acid or Pt seeds.
