# Supplementary material for: From a Chemotherapeutic Drug to a High-Performance Nanocatalyst: A Fast Colorimetric Test for Cisplatin Detection at ppb Level
Source: Biosensors (Basel). 2022 May 30;12(6):375. doi: 10.3390/bios12060375 (PMC9221495; doi:10.3390/bios12060375)
Supplement: Supplementary file 1 [file biosensors-12-00375-s001.zip › biosensors-1732164-supplementary.pdf]

Supporting Information

From a Chemotherapeutic Drug to a High-Performance Nano-catalyst: a Fast Colorimetric Test for Cisplatin Detection at ppb Level

Valentina Mastronardi [1], Mauro Moglianetti [1], Edoardo Ragusa [2], Rodolfo Zunino [2] and Pier Paolo Pompa [1,*]

[1] Nanobiointeractions&Nanodiagnostics, Istituto Italiano di Tecnologia (IIT), via Morego, 30-16163 Genova, Italy; valentina.mastronardi@iit.it (V.M.); mauro.moglianetti@icloud.com (M.M.)

[2] Department of Electrical, Electronic, Telecommunications Engineering and Naval Architecture, University of Genova, via Opera Pia, 11a-16145 Genova, Italy; edoardo.ragusa@edu.unige.it (E.R.); rodolfo.zunino@unige.it (R.Z.)

* Correspondence: pierpaolo.pompa@iit.it

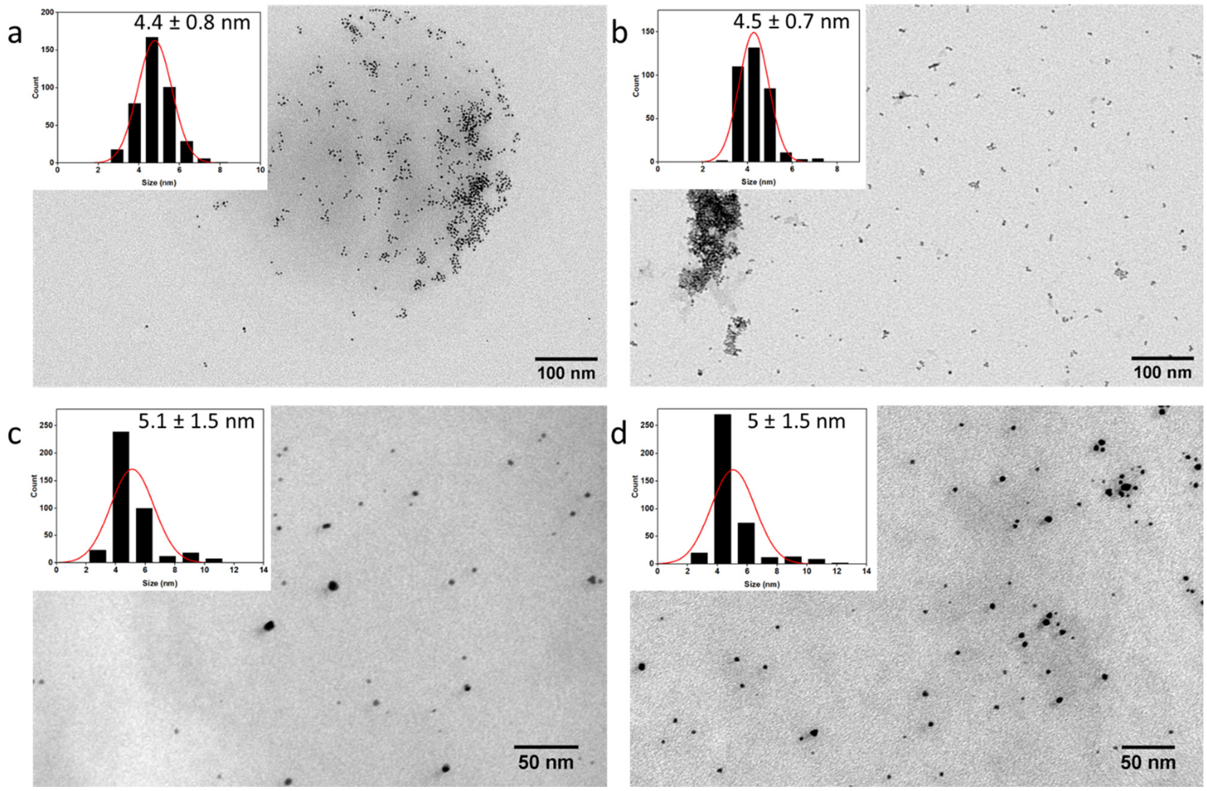

Figure S1. Representative bright-field transmission electron microscopy (BF-TEM) images and relative size distributions of PtNPs obtained from: (a) cisplatin precursor at 6.5 ppb, (b) cisplatin precursor at 45 ppb, (c) cisplatin precursor at 650 ppb, and (d) cisplatin precursor at 1000 ppb.

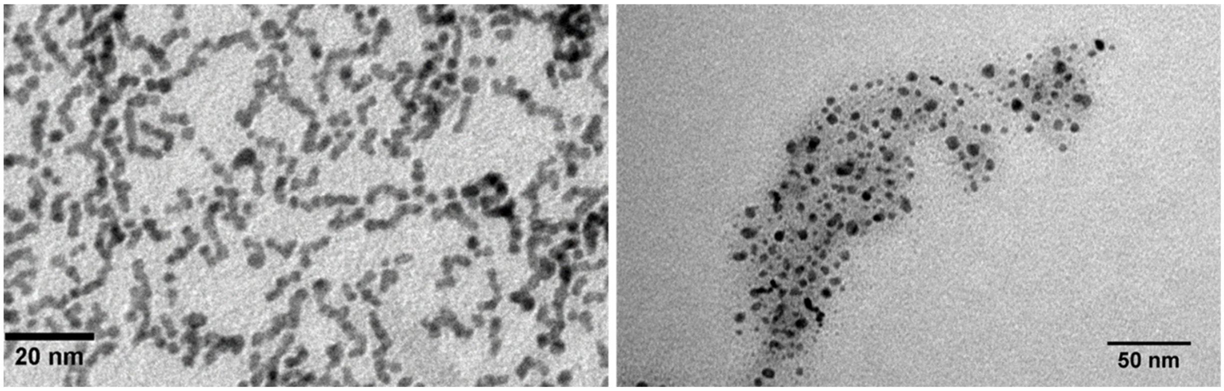

Figure S2. Representative BF-TEM images of aggregated PtNPs obtained with a high concentration $NaBH_4$ (80 mM).

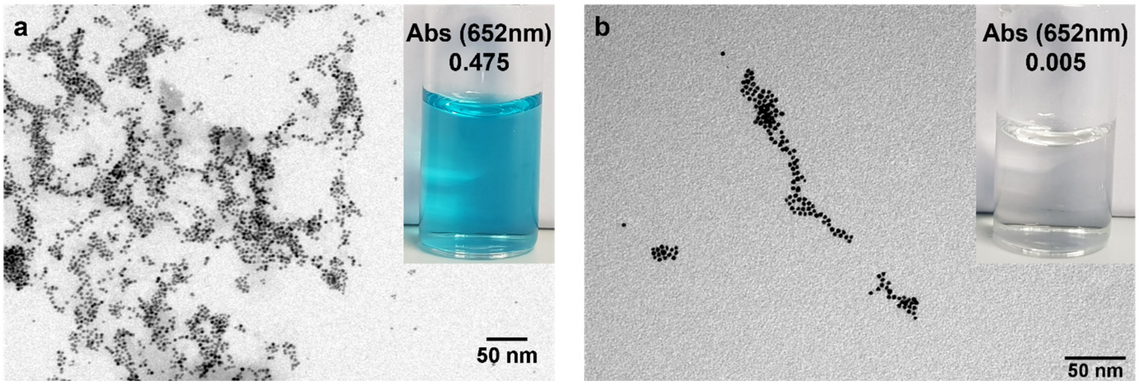

Figure S3. Comparison of the higher efficiency of cisplatin to produce PtNPs with respect to chloroplatinic acid hexahydrate (H_2PtCl_6 $6H_2O$). Bright-field transmission electron microscopy (BF-TEM) images of (a) PtNPs obtained with 230 nM (45 ppb) of cisplatin and (b) PtNPs obtained with 230 nM of chloroplatinic acid. The relative TMB assays are reported in the insets.

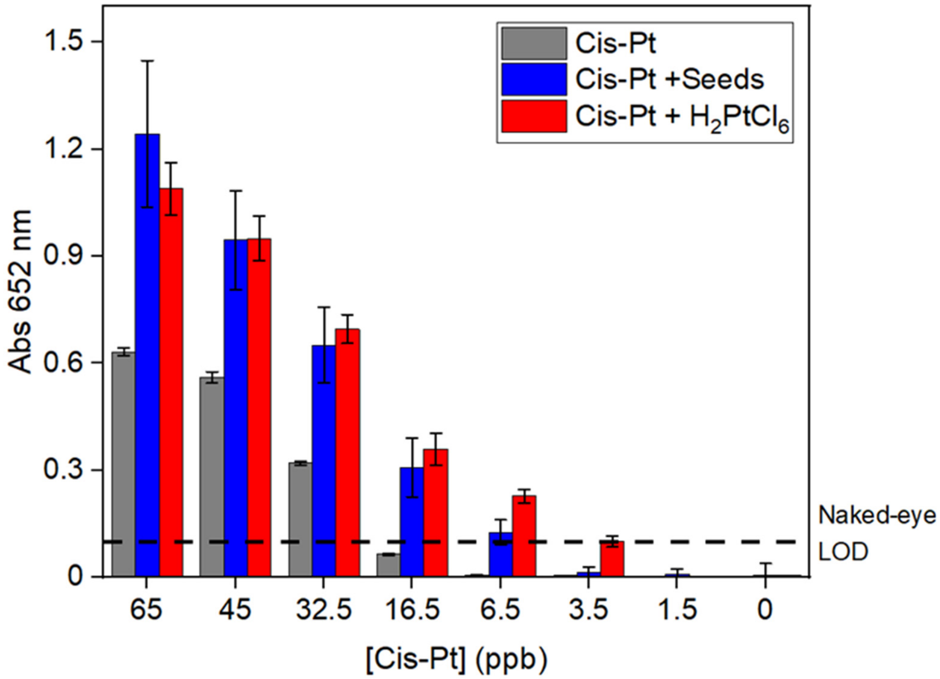

Figure S4. Sensitivity increase of the nanocatalyst-enabled colorimetric method using chloroplatinic acid (H_2PtCl_6) or Pt seeds (3 nm) as a reaction baseline, as compared to the standard configuration. The dashed line at 0.1 absorbance indicates a qualitative threshold for clear visual detection of the blue signal generated by the reaction.

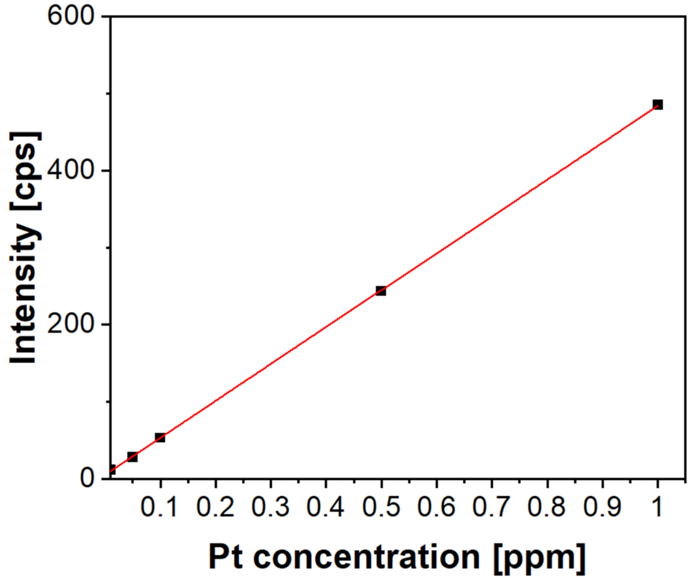

Figure S5. Detection of cisplatin by ICP-OES, showing a limit of detection (LoD) around 10 ppb.

Machine-Learning Method

Our machine-learning (ML) approach exploits the kinetic information of the colorimetric reaction. The camera films the reaction over time and stores the video. The content of the test tube is then isolated from the background, and the average value of the color is extracted for each frame generating three time series, one for each RGB channel. The consequent time series conveys all the information about the colorimetric reaction. In this framework, two test tubes containing similar cisplatin concentrations generate similar time series. ML algorithms require a systematic measure of the distance between time series. Measuring the similarity between time series is a well-known challenge that recent literature addressed in two main ways, based on the amount of available data. When a large set of data is available, deep neural networks can learn the distance function from the data [1]. When a reduced amount of data are available, predefined distance measures are preferable [2]. In our application, collecting thousands of experiments is time consuming. For this reason, we opted for a predefined distance measure. Among the existing solutions, dynamic time warping (DTW) [3,4] has proven to be an excellent solution when comparing time series characterized by different time speeds. This approach is particularly suitable in our case because high concentration of cisplatin strongly affects the speed of color change. However, to predict the concentration level of cisplatin, DTW must be coupled to a ML algorithm. In this work, DTW has been thus combined with the K-nearest neighbors (KNN) [5-7] algorithm that is the simplest solution of ML for the task of classification. Although KNN may suffer from little generalization performance when compared to other solutions, such as support vector machine (SVM) or neural networks (NN), it represents an excellent solution for small size dataset when coupled to strong distance measures like DTW. On the other hand, training and model selection for SVM and NN can become extremely challenging when only few data are available.

Using KNN with DTW as a distance measure led to a simple procedure. In the following is described the procedure for 1NN (K=1): during the training phase, the KNN algorithm creates a database saving the training data. In our case, we saved the RGB time series and the corresponding concentration levels. After the training, the algorithm can infer the cisplatin concentration in new test tubes. When a new test tube with an unknown concentration is available, the algorithm extracts the RGB time series. DTW measures the distance between the time series extracted from the new test tube and all the time series in the training set. Finally, the concentration level of the training datum with the smallest distance from the new test tube is selected. This algorithm can predict only concentration values that belong to the database; however, this is not a problem when a sufficiently large training set is available. Importantly, a dataset large enough to be used for KNN could be insufficient to be used for SVM or NN training.

References

1. Fawaz, I. H.; Forestier, G.; Weber, J.; Idoumghar, L.; Muller, P.A. Deep Learning for Time Series Classification: A Review. *Data Min. Knowl. Discov.* **2019**, *33*, 917–963, doi:10.1007/s10618-019-00619-1
2. Iwana, B.K.; Uchida, S. Time Series Data Augmentation for Neural Networks by Time Warping with a Discriminative Teacher. *Proc. Int. Conf. Pattern Recognit.* **2020**, 3558–3565, doi:10.1109/ICPR48806.2021.9412812.
3. Serrà, J.; Arcos, J.L. An Empirical Evaluation of Similarity Measures for Time Series Classification. *Knowledge-Based Syst.* **2014**, *67*, 305–314, doi:10.1016/j.knosys.2014.04.035.
4. Jing, M.; Namee, B. Mac; McLaughlin, D.; Steele, D.; McNamee, S.; Cullen, P.; Finlay, D.; McLaughlin, J. Enhance Categorisation Of Multilevel High-Sensitivity Cardiovascular Biomarkers From Lateral Flow Immunoassay Images Via Neural Networks And Dynamic Time Warping. In Proceedings of the 2020 IEEE International Conference on Image Processing (ICIP); Abu Dhabi, United Arab Emirates, 25–28 October 2020; pp. 365–369.
5. Abanda, A.; Mori, U.; Lozano, J. A. A review on distance based time series classification. *Data Min. Knowl. Discov.* **2019**, *33*, 378–412, doi:10.1007/s10618-018-0596-4
6. Bhatia, N.; Vandana. Survey of Nearest Neighbor Techniques. *arXiv* **2010**, arXiv:1007.0085
7. Younes, H.; Ibrahim, A.; Rizk, M.; Valle, M. An Efficient Selection-Based kNN Architecture for Smart Embedded Hardware Accelerators. *IEEE Open J. Circuits Syst.* **2021**, *2*, 534–545, doi:10.1109/OJCAS.2021.3108835
